# Topology Optimization of Spatially Compliant Mechanisms with an Isomorphic Matrix of a 3-UPC Type Parallel Prototype Manipulator

**DOI:** 10.3390/mi9040184

**Published:** 2018-04-14

**Authors:** Dachang Zhu, Wanghu Zhan, Fupei Wu, Alessandro Simeone

**Affiliations:** 1 School of Mechanical and Electrical Engineering, Guangzhou University, Guangzhou 510006, China; zdc98998@gzhu.edu.cn (D.Z.); cyfengyanping@gzhu.edu.cn (W.Z.); 2 Department of Mechatronic Engineering, Shantou University, Shantou 515063, China; Simeone@stu.edu.cn

**Keywords:** compliant mechanisms, topology optimization, isomorphic mapping matrix, 3-UPC type parallel prototype manipulator, solid isotropic material with penalization (SIMP)

## Abstract

A novel topology optimization approach is proposed in this paper for the design of three rotational degree-of-freedom (DOF) spatially compliant mechanisms, combining the Jacobian isomorphic mapping matrix with the solid isotropic material with penalization (SIMP) topological method. In this approach, the isomorphic Jacobian matrix of a 3-UPC (U: universal joint, P: prismatic joint, C: cylindrical joint) type parallel prototype manipulator is formulated. Subsequently, the orthogonal triangular decomposition and differential kinematic method is applied to uncouple the Jacobian matrix to construct a constraint for topology optimization. Firstly, with respect to the 3-UPC type parallel prototype manipulator, the Jacobian matrix is derived to map the inputs and outputs to be used for initializing the topology optimization process. Secondly, the orthogonal triangular decomposition with the differential kinematic method is used to reconstruct the uncoupled mapping matrix to derive the 3-UPC type parallel prototype manipulator. Finally, a combination of the solid isotropic material with penalization (SIMP) method and the isomorphic mapping matrix is applied to construct the topological model. A typical three rotational DOF spatially compliant mechanism is reported as a numerical example to demonstrate the effectiveness of the proposed method.

## 1. Introduction

Compliant mechanisms are widely used in precision manufacturing as they do not show problems such as clearance, friction and wear [1]. In the past two decades, flexible hinges have been replacing the rigid hinges in parallel mechanism configurations, and the pseudo rigid body method has been used to build flexible or compliant mechanism models [2,3,4]. However, the whole structural stiffness maximization is not satisfied by this method, while compliant mechanisms are limited in use to micro positioning due to their spatial multi-DOF (degree-of-freedom) motion characteristics [5,6,7]. Recently, flexible beam structures and flexure joints are used to overcome these issues [8,9,10,11], such as Tan et al. [12], who proposed a multi-leaf configuration of expanded-motion-range flexure hinges.

Previous works [13,14,15,16] highlighted how research into compliant mechanisms is divided into two main directions: large stroke with multi-DOF and micro/nano scale displacement with multi-DOF. The latter scope is further divided into lumped compliance (often referred to as the pseudo-rigid-body-model, PRBM) and distributed compliance (often referred to as topology optimization), as reported in [17]. This paper focuses on topology optimization.

Considering the multi-DOF motion characteristics of parallel manipulators, the hinge-replacing method is used to reconstruct the spatially compliant mechanism. Examples can be found in the 6-DOF compliant parallel mechanisms designed by Hudgens et al. [18] and Mclnroy et al. [19] and in the three-DOF planar-compliant parallel mechanisms proposed by Yi et al. [20] and Hao et al. [21]. The model of compliant mechanisms with flexure joints can be built by using the pseudo-rigid-body approach in contrast to the original structure of parallel manipulators. To improve the overall stiffness of compliant mechanisms, the topology optimization approach has been used for its design. For example, Sigmund [22,23,24] developed the density method based on the continuum-type topology optimization technique for the optimal design of compliant mechanisms. Using the mutual energy approach, the homogenization method is adopted by Nishiwaki [25] to solve compliant mechanism optimization problems, and Saxena [26] generalized multi-criteria formulations for the performance improvement of output deformation and strain energy computation. According to the stiffness of elements, Wang [27] proposed a linear elastic structural analysis method. Large displacement, multiple materials, and 3D topology optimization methods for compliant mechanisms are proposed in [28,29,30].

In hingeless topological structures, the pseudo-rigid-body method is not suitable for modeling, because there is no exact rigid mechanism corresponding to the topological structure, moreover, the error related to flexible elements is reported in the literature [31,32,33]. To overcome this shortcoming, the Jacobian matrix of spatial multi-DOF parallel manipulators may be considered during the topology optimization process. The Jacobian matrix is the mapping relationship between the operation space and the joint space, and it is only concerned with structural parameters. A Jacobian matrix based on a compliance frame is adopted in [34,35], and a planar-compliant mechanism is proposed. However, the compliance frame is changed during topology optimization process, thus it cannot be used to model the topological structure of the proposed planar-compliant mechanism. In this paper, a novel topology optimization method for a three-DOF spatially compliant mechanism is proposed by combining the Jacobian isomorphic mapping matrix with solid isotropic material with penalization (SIMP) topology optimization method.

The paper is organized as follows. By using the differential approximation method, the Jacobian isomorphic mapping matrix of the 3-UPC (U: universal joint, P: prismatic joint, C: cylindrical joint) type parallel prototype manipulator is calculated in Section 2. Section 3 reports the Jacobian isomorphic mapping matrix combination with SIMP, along with the topological model of spatial compliant mechanism with three rotational DOF. The sensitivities analysis and topological structure derivation are described in Section 4. Experimental investigations are shown in Section 5. Concluding remarks are summarized in Section 6.

## 2. Isomorphic Mapping Matrix of the 3-UPC Type Parallel Prototype Manipulator

**Figure 1:**
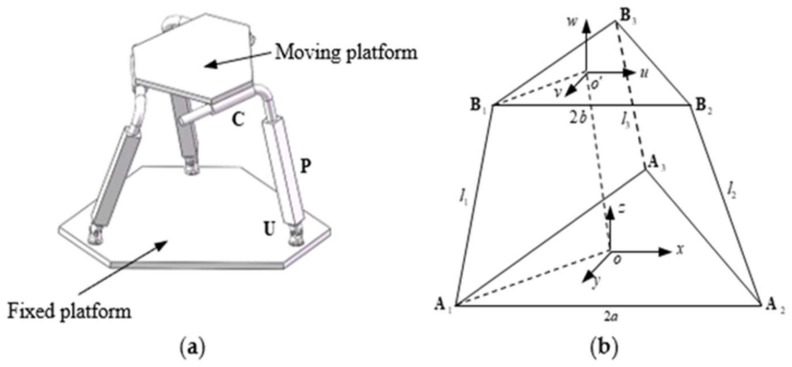
3-UPC type parallel prototype manipulator (**a**) structure configuration model, (**b**) parameters with coordinate frame.

The 3-UPC type parallel prototype manipulator is composed of a moving platform, fixed platform and three symmetrical links [36]. Each link is composed by a universal joint (U) that is connected to the fixed platform, a cylindrical joint (C) that is connected to the moving platform, and an actuated prismatic joint (P) that is used to connect the universal joint with the cylindrical joint. Its structural configuration is shown in Figure 1.

The {o−xyz} coordinate system is fixed to the base platform, and the $\left\{o^{\prime }-uvw\right\}$ coordinate system is fixed to the moving platform. The connection point of the $i^{th}$ limb (sub-chain) lying on the base platform is denoted by vector $A_{i}={\left(a_{x}^{i},a_{y}^{i},a_{z}^{i}\right)}^{T}$, i=1,2,3, and the connection point of $i^{th}$ limb (sub-chain) lying on the moving platform is denoted by vector $B_{i}={\left(b_{x}^{i},b_{y}^{i},b_{z}^{i}\right)}^{T}$, i=1,2,3. The limbs are of equal initial length, their value is constant and equal to l, which means $l_{1}=l_{2}=l_{3}=l$. The upper and base platforms are in the shape of an equilateral triangle, and the lengths of the upper and base platforms are 2b and 2a, respectively.

The kinematic properties of the 3-UPC type parallel manipulator have been analyzed by means of screw theory in [37]. The compliant mechanism obtained by the topology optimization method is characterized by a micro/nanoscale motion. If factors such as friction and joint clearance are not taken into account, theoretically the parallel prototype mechanism could achieve the same micro/nanoscale motion as the compliant mechanism. Under these circumstances, it is likely that the motion range of each motion branch in the parallel prototype will tend to be infinitesimal. Assuming that the three kinematic rotational parameters were α,β,γ, and that the initial topological convergence conditions were set as 1 × 10^−6^ rad, without loss of generality, the following approximation was adopted: sin(α)=α and cos(α)=1. As the product of two infinitesimal quantities is a high order infinitesimal, we can assume that α⋅β=α⋅γ=β⋅γ=0. The above hypothesis applies equally to γ and β.

According to the Euler method and assumptions, the rotational transformation matrix is given by

(1) $$R\left(\alpha ,\beta ,\gamma \right)=R_{x}\left(\alpha \right)R_{y}\left(\beta \right)R_{z}\left(\gamma \right)=\left[\begin{array}{ccc} c\beta c\gamma & -c\beta s\gamma & s\beta \\ -s\alpha s\beta +c\alpha s\gamma & -s\alpha s\beta s\gamma +c\alpha c\gamma & -s\alpha c\beta \\ c\alpha s\beta c\gamma +s\alpha s\gamma & -c\alpha s\beta s\gamma +s\alpha c\gamma & c\alpha c\beta \end{array}\right]=\left[\begin{array}{ccc} 1 & -\gamma & \beta \\ \gamma & 1 & -\alpha \\ -\beta & \alpha & 1 \end{array}\right]$$

where sin(⋅)=s⋅ and cos(⋅)=c⋅.

Assuming that the driving force is provided by peristaltic joint, and the value of actuated is $\Delta l_{i}$, i=1,2,3, the vector equations can be written as follows

(2) $$\vec{A_{i}o}+\vec{oo^{\prime }}=\vec{o^{\prime }{B^{\prime }}_{i}}+\vec{{B^{\prime }}_{i}A_{i}},\;i=1,2,3$$

Multiplying the rotational transformation matrix by the original coordinate frame of ${B^{\prime }}_{i}$, the coordinate frame of ${B^{\prime }}_{i}$ is derived as

(3) $${B^{\prime }}_{i}=B_{i}R\left(\alpha ,\beta ,\gamma \right),\;i=1,2,3$$

Substituting the original coordinate frames into Equation (3), the isomorphic mapping matrix can be written as follows

(4) $$\left[\begin{array}{c} \Delta l_{1}\\ \Delta l_{2}\\ \Delta l_{3} \end{array}\right]=\left[\begin{array}{ccc} \frac{\sqrt{3}ah}{3l^{2}} & \frac{2bh}{l^{2}} & \frac{2\sqrt{3}\left(a-b\right)b}{3l^{2}}\\ -\frac{\sqrt{3}ah}{3l^{2}} & \frac{ah}{l^{2}} & 0\\ \frac{2\sqrt{3}ah}{3l^{2}} & 0 & 0 \end{array}\right]\left[\begin{array}{c} \alpha \\ \beta \\ \gamma \end{array}\right]=J_{D}\left[\begin{array}{c} \alpha \\ \beta \\ \gamma \end{array}\right]$$

where $J_{D}$ is the Jacobian isomorphic mapping matrix used for the topology optimization process.

## 3. Topology Optimization Model of the Three-DOF Spatially Compliant Mechanism

### 3.1. Model of the Solid Isotropic Material with Penalization (SIMP)

Combining the finite element method with the optimization method, topology optimization is used to solve the material distribution problem in the area of a given design field according to the load or support constraints. The SIMP method is proposed on the basis of the relative density method, and the optimization process is determined by the unit selection and relative density with a penalty factor [25].

By introducing a penalty factor, a dominant nonlinear correspondence is established between the elastic modulus of the material and the relative density of the element, which makes the middle density value equal to 0 or 1. The relationship between the element density $x_{i}$ and element Young’s modulus $E_{i}$ can be written as follows

(5) $$E_{i}=E_{i}\left(x_{i}\right)=x_{i}^{p}E_{0},\;x_{i}\in \left[0,1\right]$$

where $E_{0}$ is the elastic modulus of the solid material. p is the penalty factor of the intermediate material density, and its values range can be defined as

(6) $$p\geq max\left\{\frac{2}{1-v^{0}},\frac{4}{1+v^{0}}\right\}$$

where $v^{0}$ is the Poisson’s ratio.

The optimal model of the three-rotational DOF spatially compliant mechanism is described as follows

(7) $$\begin{array}{l} {\begin{array}{cc} find & x=\left[x_{1},x_{2},\cdots x_{n}\right] \end{array}}^{T}\\ \begin{array}{cc} min & c\left(x\right)=F^{T}U\left(x\right)=\sum_{i=1}^{3}\sum_{j=1}^{3}{\tilde{U}}_{j}^{T}\left(x\right) \end{array}K\left(x\right)U_{i}\left(x\right)=\sum_{e=1}^{n}\sum_{i=1}^{3}\sum_{j=1}^{3}{\tilde{U}}_{ej}^{T}\left(x_{j}\right)\rho_{e}^{p}K_{e}^{ij}U_{ei}\left(x_{i}\right)\\ \begin{array}{cc} s.t. & v\left(x\right)=x^{T} \end{array}\cdot v-\bar{v}\\ \begin{array}{cc} & \;J_{D}\cdot U=L \end{array}\\ \begin{array}{cc} & \;K\left(x\right) \end{array}\cdot U\left(x\right)=F\\ \begin{array}{cc} & \;x\in R^{n} \end{array},0\leq x\leq 1 \end{array}$$

where x is the density function, defined as $x_{i}=\frac{\sum_{j\in n}H_{ij}v_{j}x_{j}}{\sum_{j\in n}H_{ij}v_{j}}$, j is the neighborhood of an element $x_{i}$ with volume $v_{i}$, $H_{ij}$ is a weight factor, n is the number of elements, $v={\left[v_{1},v_{2}\cdots v_{n}\right]}^{T}$ is element volume, $\bar{v}$ is the convergence objective of the designed volume. The nodal force vector F is the input force vectors, and U(x) is the output with displacement vector. By using Equation (4), the displacement with input L and output U is the solution of $J_{D}\cdot U=L$. K is the whole stiffness of the compliant mechanism, and $U_{ei}$ is the $i^{th}$ element of the displacement vector, ${\tilde{U}}_{ej}$ is the $j^{th}$ element of the displacement vector. $K_{e}$ is the stiffness of the element and $\rho_{e}$ is the density of the element.

### 3.2. Sensitivity Analysis

Using the sensitivity values of the objective and constraint functions, the change of direction of the design variables during the topology optimization process can be determined.

Deriving the volume constraint v(x) in Equation (7) with respect to the design variable $x_{e}$, yields to

(8) $$\frac{\partial v\left(x\right)}{\partial x_{e}}=\sum_{i\in n}\frac{\partial v\left(x\right)}{\partial x_{i}}\frac{\partial x_{i}}{\partial x_{e}}$$

where $\frac{\partial v\left(x\right)}{\partial x_{i}}=v_{i}$ and $\frac{\partial x_{i}}{\partial x_{e}}=\frac{H_{ie}v_{e}}{\sum_{j\in n}H_{ij}v_{j}}$.

A hexahedral element structure was adopted to mesh the partition grid, that is, $v_{i}=v_{j}=v_{e}=1$.

The compliance sensitivity is satisfied by the following equation

(9) $$\frac{\partial c\left(x\right)}{\partial x_{i}}=F^{T}\frac{\partial U\left(x\right)}{\partial x_{i}}=U{\left(x\right)}^{T}K\left(x\right)\frac{\partial U\left(x\right)}{\partial x_{i}}$$

where the nodal displacement vector U(x) is the solution of the equilibrium equation, K(x)U(x)=F, and its derivative with respect to $x_{i}$ is satisfied by

(10) $$\frac{\partial K\left(x\right)}{\partial x_{i}}+K\left(x\right)\frac{\partial U\left(x\right)}{\partial x_{i}}=0$$

Which yields

(11) $$\frac{\partial U\left(x\right)}{\partial x_{i}}=-K{\left(x\right)}^{-1}\frac{\partial K\left(x\right)}{\partial x_{i}}U\left(x\right)$$

The element stiffness matrices $K_{i}$ and $K_{i}^{0}$ are used to satisfy Equation (12)

(12) $$K\left(x\right)=\sum_{i=1}^{n}K_{i}\left(x_{i}\right)=\sum_{i=1}^{n}E_{i}\left(x_{i}\right)K_{i}^{0}$$

then,

(13) $$\frac{\partial K\left(x\right)}{\partial x_{i}}=\frac{\partial }{\partial x_{i}}\sum_{i=1}^{n}x_{i}^{p}E_{0}K_{i}^{0}=px_{i}^{p-1}E_{0}K_{i}^{0}$$

Equation (11) can be rewritten as

(14) $$\frac{\partial c\left(x\right)}{\partial x_{i}}=-U{\left(x\right)}^{T}px_{i}^{p-1}E_{0}K_{i}^{0}U\left(x\right)$$

Since $K_{i}^{0}$ is element stiffness matrix, Equation (14) can be denoted as

(15) $$\frac{\partial c\left(x\right)}{\partial x_{i}}=-u_{i}{\left(x\right)}^{T}px_{i}^{p-1}E_{0}k_{i}^{0}u_{i}\left(x\right)$$

where $u_{i}$ is the nodal displacement vector element, and $K_{i}^{0}$ is positive definite, then

(16) $$\frac{\partial c\left(x\right)}{\partial x_{i}}<0$$

The derivative of the objective function with the design variables is

(17) $$\begin{array}{c} \frac{\partial c\left(x\right)}{\partial \rho_{e}}=\frac{\partial \left(\sum_{i=1}^{3}\sum_{j=1}^{3}{\tilde{U}}_{j}^{T}\left(x\right)KU_{i}\left(x\right)\right)}{\partial \rho_{e}}=\left(\frac{\partial \sum_{j=1}^{3}{\tilde{U}}_{j}^{T}\left(x\right)}{\partial \rho_{e}}K+\sum_{j=1}^{3}{\tilde{U}}_{j}^{T}\left(x\right)\frac{\partial K}{\partial \rho_{e}}\right)\sum_{i=1}^{3}U_{i}\left(x\right)+\\ \sum_{j=1}^{3}{\tilde{U}}_{j}^{T}\left(x\right)\left(\frac{\partial K}{\partial \rho_{e}}\sum_{i=1}^{3}U_{i}\left(x\right)+K\frac{\partial \sum_{i=1}^{3}U_{i}\left(x\right)}{\partial \rho_{e}}\right)-\sum_{j=1}^{3}{\tilde{U}}_{j}^{T}\left(x\right)\frac{\partial K}{\partial \rho_{e}}\sum_{i=1}^{3}U_{i}\left(x\right) \end{array}$$

Without the loss of generality, input load independence from design variables was assumed. Differentiating the design variables of Equation (7) with respect to the stiffness matrix, yields

(18) $$\frac{\partial K}{\partial \rho_{e}}\sum_{j=1}^{3}{\tilde{U}}_{j}^{T}\left(x\right)+K\frac{\partial \sum_{j=1}^{3}{\tilde{U}}_{j}^{T}\left(x\right)}{\partial \rho_{e}}=0\;\mathrm{and}\;\frac{\partial K}{\partial \rho_{e}}\sum_{i=1}^{3}U_{i}^{T}\left(x\right)+K\frac{\partial \sum_{i=1}^{3}U_{i}^{T}\left(x\right)}{\partial \rho_{e}}=0$$

Substituting $K=\sum_{e=1}^{n}\rho_{e}^{p}k_{e}$ into Equation (18) and using Equation (17), the sensitivity of the objective function can be given by

(19) $$\frac{\partial c\left(x\right)}{\partial \rho_{e}}=\sum_{e=1}^{n}\sum_{i=1}^{3}\sum_{j=1}^{3}p{\tilde{U}}_{j}^{T}\left(x\right)\rho_{e}^{p-1}k_{e}U_{i}\left(x\right)$$

With the volume as the optimization constraint, the partial derivative of the volume with respect to the material density is given by the constraint function sensitivity:

(20) $$\frac{\partial v\left(x\right)}{\partial \rho_{e}}=\frac{\partial \left(\int_{\Omega }\rho_{e}dv\right)}{\partial \rho_{e}}=1$$

### 3.3. Solution of the Topology Optimization Model

Based on the variation principle, the function equation that contains the compliance, objective, and constraint functions can be built using the Lagrange multiplier. The update iteration of the multi-input–output in the three-dimensional space is given by:

(21) $$\rho_{e}^{\left(k+1\right)}=\left\{ \begin{array}{l} \min\{(m+1)\rho_{e}^{\left(k\right)},1\}\;if\;min\{(1+m)\rho_{e}^{\left(k\right)},1\}\leq (D_{e}^{\left(k\right)}{)}^{\varsigma }\rho_{e}^{\left(k\right)}\\ (D_{e}^{\left(k\right)}{)}^{\varsigma }\rho_{e}^{\left(k\right)}\;if\;\max\{(1-m)\rho_{e}^{\left(k\right)},\rho_{\min}\}<(D_{e}^{\left(k\right)}{)}^{\varsigma }\rho_{e}^{\left(k\right)}\\ \;<\min\{(1+m)\rho_{e}^{\left(k\right)},1\}\\ \max\{(1-m)\rho_{e}^{\left(k\right)},\rho_{\min}\}\;if\;(D_{e}^{\left(k\right)}{)}^{\varsigma }\rho_{e}^{\left(k\right)}\leq \max\{(1-m)\rho_{e}^{\left(k\right)},\rho_{\min}\} \end{array}\right.$$

where $\rho_{e}^{\left(k\right)}$ is the iteration value of $k^{th}$ step, $\rho_{e}^{\left(k+1\right)}$ is the iteration value of $k+1^{th}$ step, *m* is the moving limit constant (*m* = 0.1~0.3) and ς is the damping factor (ς = 0.4~0.5).

$D_{e}^{\left(k\right)}$ is expressed as follows:

(22) $$D_{e}^{\left(k\right)}=\frac{p\rho_{e}^{p-1}\sum_{j=1}^{3}{\tilde{U}}_{j}^{T}(x)KE\sum_{i=1}^{3}U_{i}(x)\Delta E}{\Lambda^{\left(k\right)}V_{e}}=(max(0,-\frac{\partial c(x)}{\partial \rho_{e}}))/\Lambda^{\left(k\right)}V_{e}$$

$\Lambda^{\left(k\right)}$ is the Lagrange multiplier with the volume constraint of $k^{\mathrm{th}}$ step iteration, updated adopting bi-directional convex linear programming method, and $v_{e}$ is the element volume of $k^{\mathrm{th}}$ step iteration.

## 4. Topology Structure of the Three-DOF Spatially Compliant Mechanism

### 4.1. Design Domain and Iterative Algorithm

**Figure 2:**
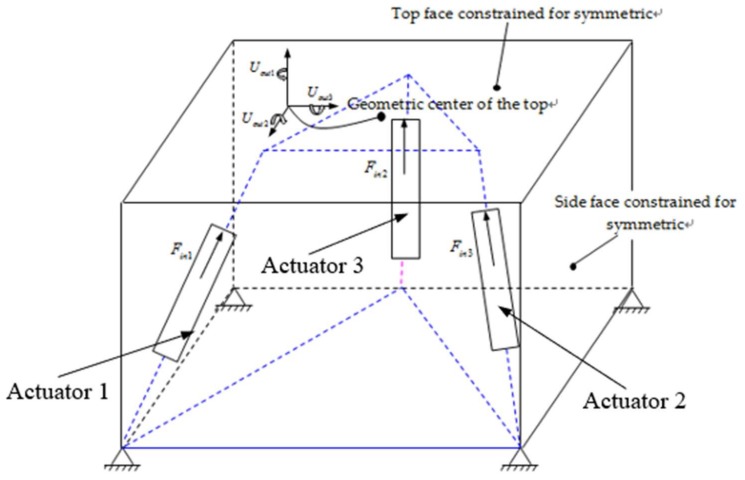
Initial design domain of the three-DOF spatially compliant mechanism.

In this section, the design of three-dimensional space actuators is carried out, and in all cases boundary conditions forcing the treatment of the design object as a three-dimensional problem are adopted. Three directions of actuating displacement are used to design the three-DOF spatially compliant mechanism. The initial design domain is shown in Figure 2, where the dimensions are also indicated.

The domain is a cube of dimension 125 mm × 125 mm × 125 mm discretized with 2000 eight node elements. In order to reduce analysis computation time, the Hypermesh^@^ software (version 2017, Altair Engineering, Troy, MI, USA) was used to mesh the partition. In this case it is assumed that the three-DOF spatially compliant mechanism will consist of a material with a Young’s modulus 2.06 × 10^−5^ F/mm^2^ and Poisson’s ratio 0.3. Considering the literature [38] and the actuated configuration of a parallel prototype manipulator, the design three-DOF spatially compliant mechanism is set to three actuated loads of $F_{in1}=F_{in2}=F_{in3}=1\;N$, and three constraint displacements are designed at the center point of the top domain. The volume restriction is 20 percent of the design domain. For the elastic workpiece, a value of 2 × 10^6^ mN/m is assigned to the spring constant.

The iterative algorithm of topology optimization for the three-DOF spatially compliant mechanism is given as follows.

Step 1: The hexahedral mesh method is used to divide the design region of Figure 2 into grids, and the size of the grid is determined.

Step 2: According to the spatially geometric constraint of a parallel prototype manipulator of the 3-UPC type, the boundary constraints and load conditions are defined, and combined with the SIMP topology optimization method, proposed in Equation (7).

Step 3: Initial values are assigned to the design variables.

Step 4: Equation (21) is differentiated with the flexibility function.

Step 5: The sensitivity of the constraint function is calculated using Equation (7), and the sensitivities of the material density and design variables are derived by Equation (18) and (20), respectively.

Step 6: The optimization criteria denoted by Equation (21) are calculated, and the design variables are updated as in Equation (22). The mesh elements of the design domain will be removed if they satisfy Equation (16).

Step 7: The optimization process will terminate when the remaining elements satisfy the volume constraint in Equation (8) and (17). Otherwise, the process will return to Step 4.

### 4.2. The Topology Optimization Structure of the Three-DOF Spatially Compliant Mechanism

**Figure 3:**
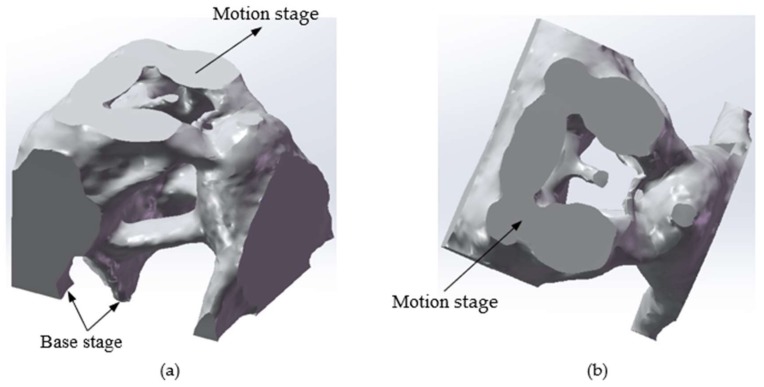
Topology optimization structure of the three-DOF spatially compliant mechanism, (**a**) Structure of the three-DOF spatially compliant mechanism; (**b**) Top view of this structure.

**Figure 4:**
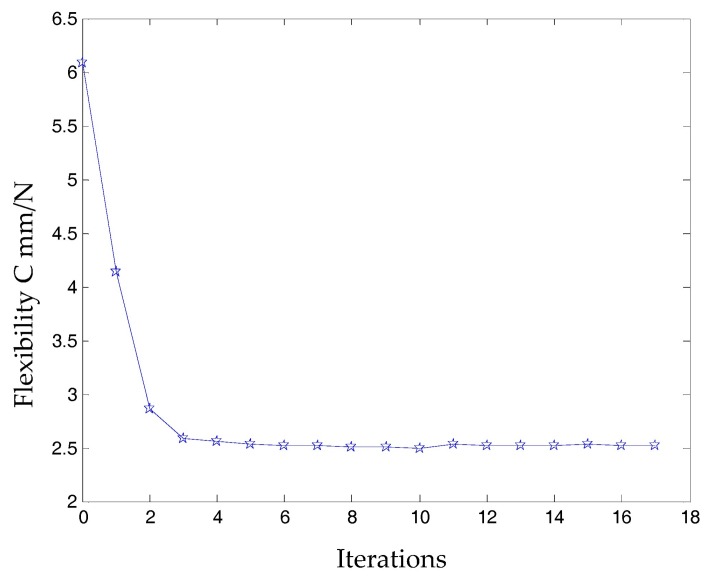
Topology optimization iteration procedure of the three-DOF spatially compliant mechanism.

The topology optimization structure of the three-DOF spatially compliant mechanism with an isomorphic mapping matrix can be obtained as shown in Figure 3. The topology optimization iteration procedure of the three-DOF spatially compliant mechanism is shown in Figure 4 and converges after four iterative steps.

## 5. Numerical Implementation

### 5.1. Analysis of Differential Kinematic Characteristics

The differential kinematic approximation method is used to derive the isomorphic mapping matrix as reported in Section 2. Furthermore, this isomorphic mapping matrix is applied to the topology optimization process as a constraint condition. The SimMechanical^@^ software (version 2013a, MathWorks, Inc., Natick, MA, USA) is adopted to build a model of the 3-UPC type parallel prototype manipulator under the initial structural conditions that are shown as Table 1.

**Figure 5:**
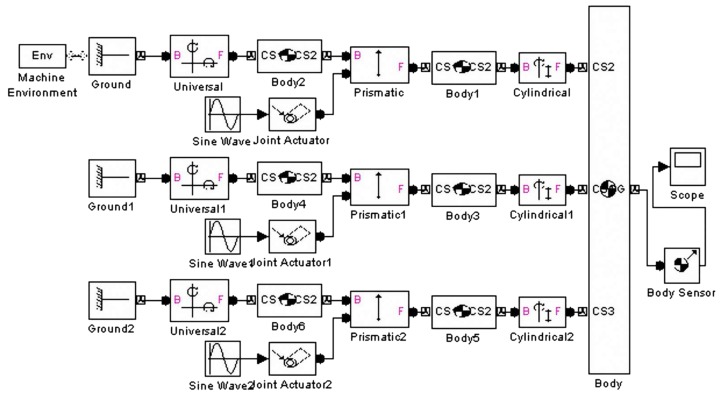
Structure of the 3-UPC type parallel prototype manipulator using SimMechanical^@^ software.

The structure of the 3-UPC type parallel prototype manipulator is shown in Figure 5.

Substituting the structural initial parameters into Equation (4), the isomorphic mapping matrix can be given by

(23) $$\left[\begin{array}{c} \Delta l_{1}\\ \Delta l_{2}\\ \Delta l_{3} \end{array}\right]=\left[\begin{array}{ccc} 0.404 & 0.932 & 0.135\\ -0.404 & 0.7 & 0\\ 0.81 & 0 & 0 \end{array}\right]\left[\begin{array}{c} \alpha \\ \beta \\ \gamma \end{array}\right]$$

**Figure 6:**
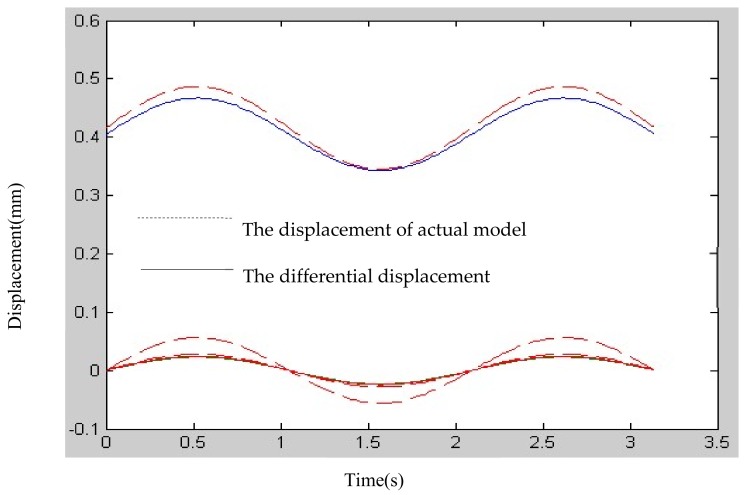
Forward kinematic comparison between differential kinematic approximation method and actual model of the 3-UPC type parallel prototype manipulator.

Figure 6 shows that the isomorphism mapping matrix obtained using differential kinematic approximation methods maintains the isomorphic kinematic characteristics of the 3-UPC type parallel prototype manipulator, and its kinematic errors corresponding to small signal inputs are 0.34%, 0.56% and 1.56%, respectively.

### 5.2. Differential Kinematic Characteristics of the Topology Optimization Structure

**Figure 7:**
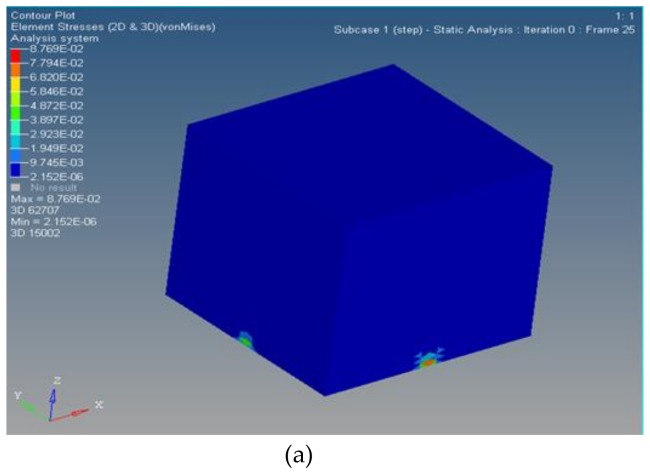

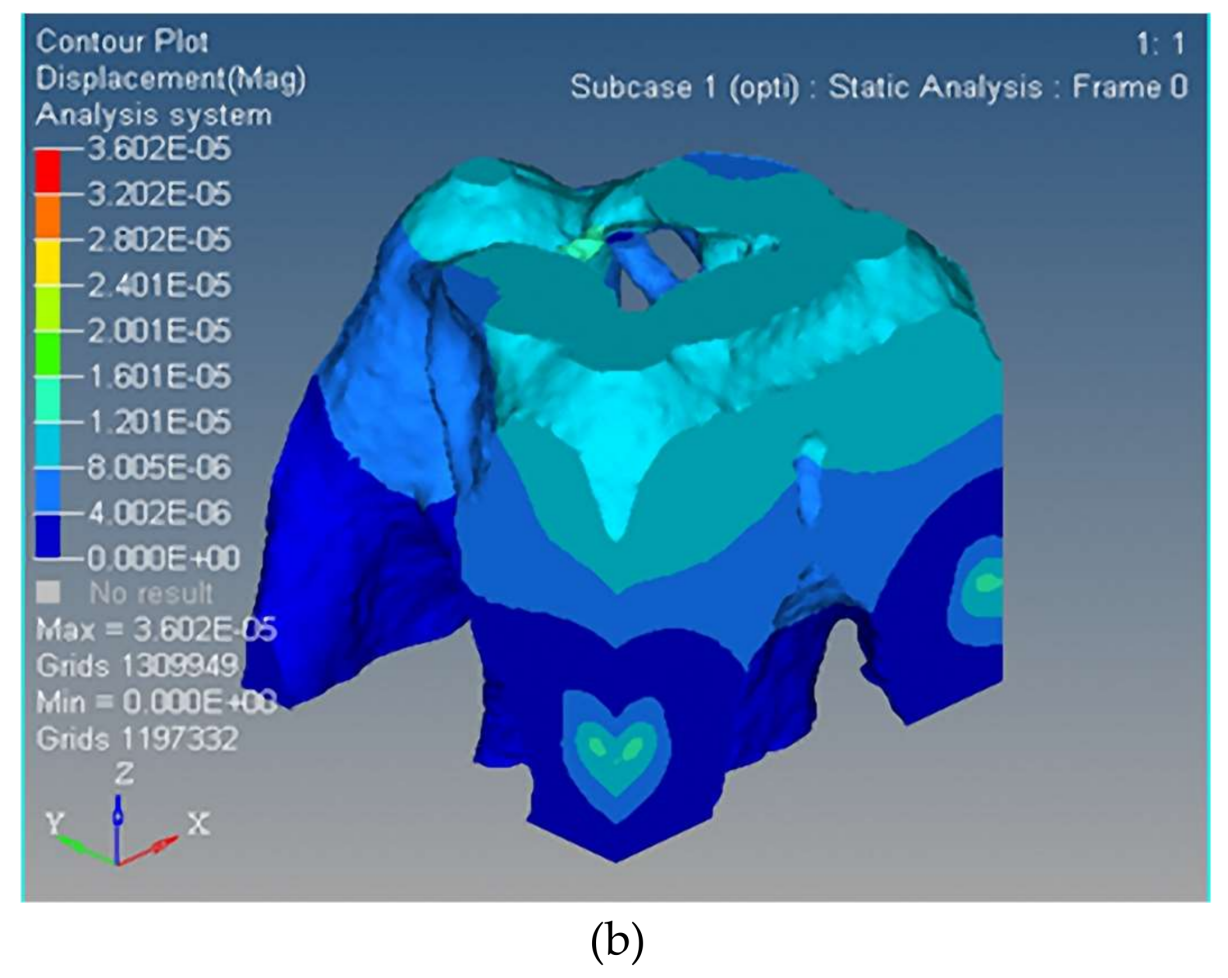
Stress distribution comparison: (**a**) before optimization; (**b**) after optimization.

The stress distribution values before and after topology optimization are reported in Table 2 and illustrated in Figure 7.

**Figure 8:**
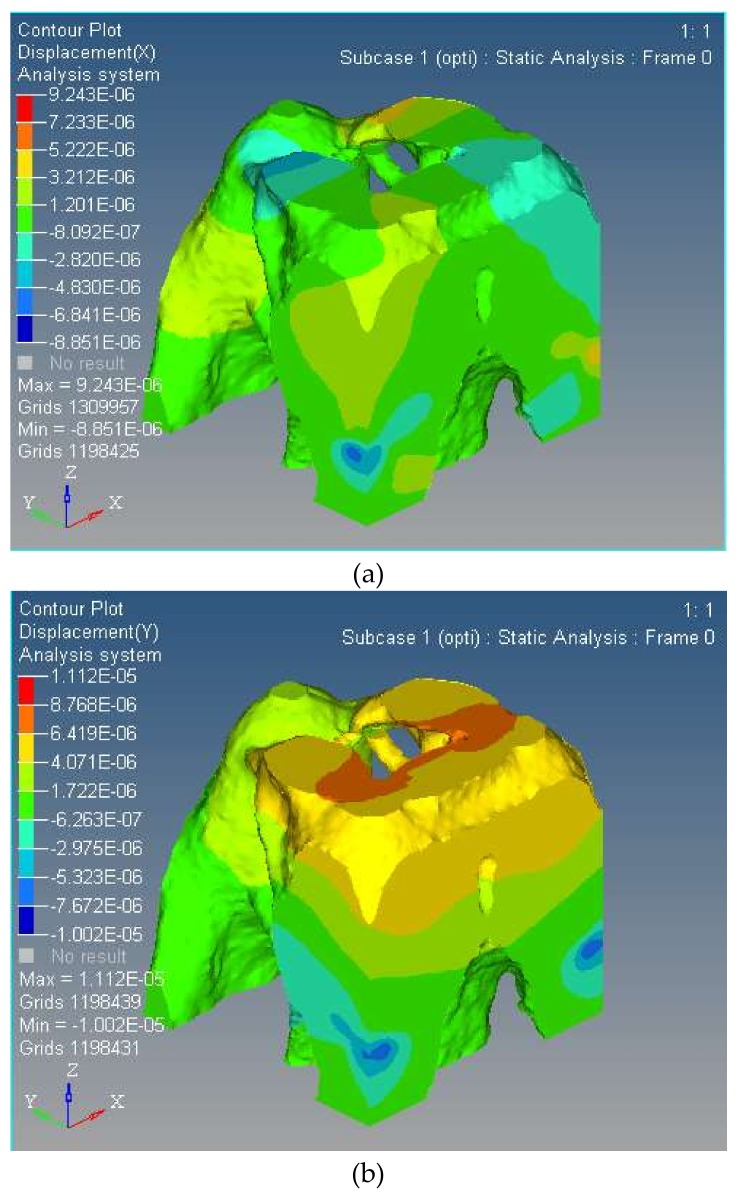

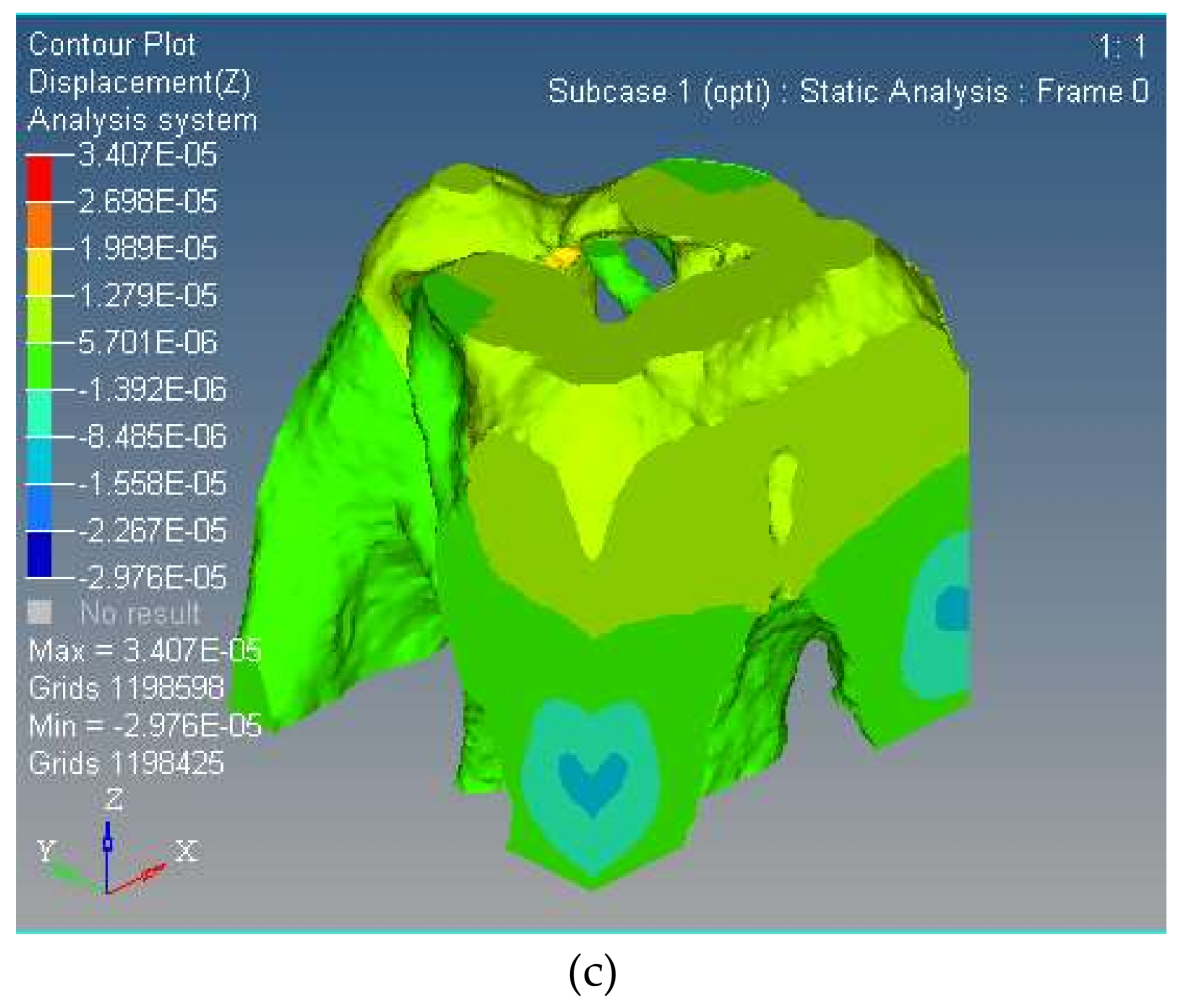
The three nano-scale-rotational displacements of the spatially compliant mechanism; (**a**) Along the x-axis the maximum displacement is 7.774 × 10^−7^ rad and the minimum displacement is −6.299 × 10^−7^ rad; (**b**) Along the y-axis the maximum displacement is 3.541 × 10^−6^ rad and the minimum displacement is −2.299 × 10^−7^ rad; (**c**) Along the z-axis the maximum displacement is 4.344 × 10^−6^ rad and the minimum displacement is 0 rad.

The topology optimization structure of three-DOF spatially compliant mechanism is shown in Figure 3, and its three nano-rotational direction displacements are shown as Figure 8.

### 5.3. Experimental Study

Before the optimization, a background hexahedron mesh was set up for the reference domain. In each iteration step of the optimization process, the modification of the fixed background mesh was performed to obtain a mesh that is conformal to the geometry of the compliant mechanism. On the other hand, in order to place the piezoelectric (PZT) actuator, three non-design areas were set according to the driver configuration characteristics of the parallel prototype manipulator. The output displacements corresponding to the center of the top platform were measured by Renishaw laser interferometer, and the experiments were conducted under a closed-loop control strategy by using a proportional-integral-derivative (PID) controller. The parameters of three PZT actuators are given in Table 3.

**Figure 9:**
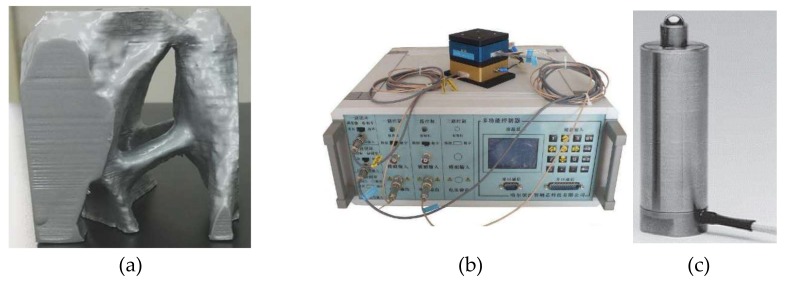
Topological structure, controller and actuator. (**a**) The physical model of the topological structure, (**b**) Controller, (**c**) PZT actuator.

The topological structure, controller and actuator are shown in Figure 9. A formula for the calculation of voltage and output of the PZT actuators is used:

(24) $$M_{piezo}^{active}(t)=\frac{b\cdot E_{p}\cdot (t_{b}+t_{p})}{2}\cdot V_{actuator}(t)=M_{p}\cdot V_{actuator}(t)$$

where $M_{piezo}^{active}(t)$ is the output of PZT actuators, $E_{p}$ is electrostatic capacity, $M_{p}$ is the constant parameter, b is the width of PZT actuator, $t_{b}$ is the installation channel, $t_{p}$ is the thickness of PZT, and $V_{actuator}(t)$ is the applied voltage.

**Figure 10:**
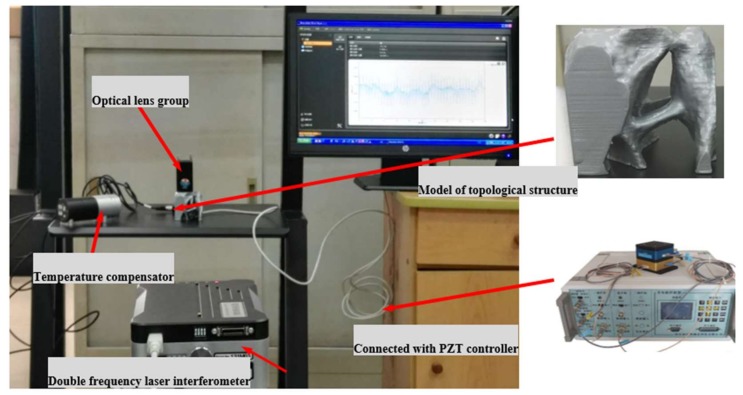
Experimental device.

The experimental device is shown in Figure 10. The nominal stroke is the displacement stroke corresponding to the driving voltage of 0–150 V; for high reliability long term use, the recommended driving voltage is 0–120 V, and the maximum output up to 25,000N.

### 5.4. Experimental Results and Analysis

**Figure 11:**
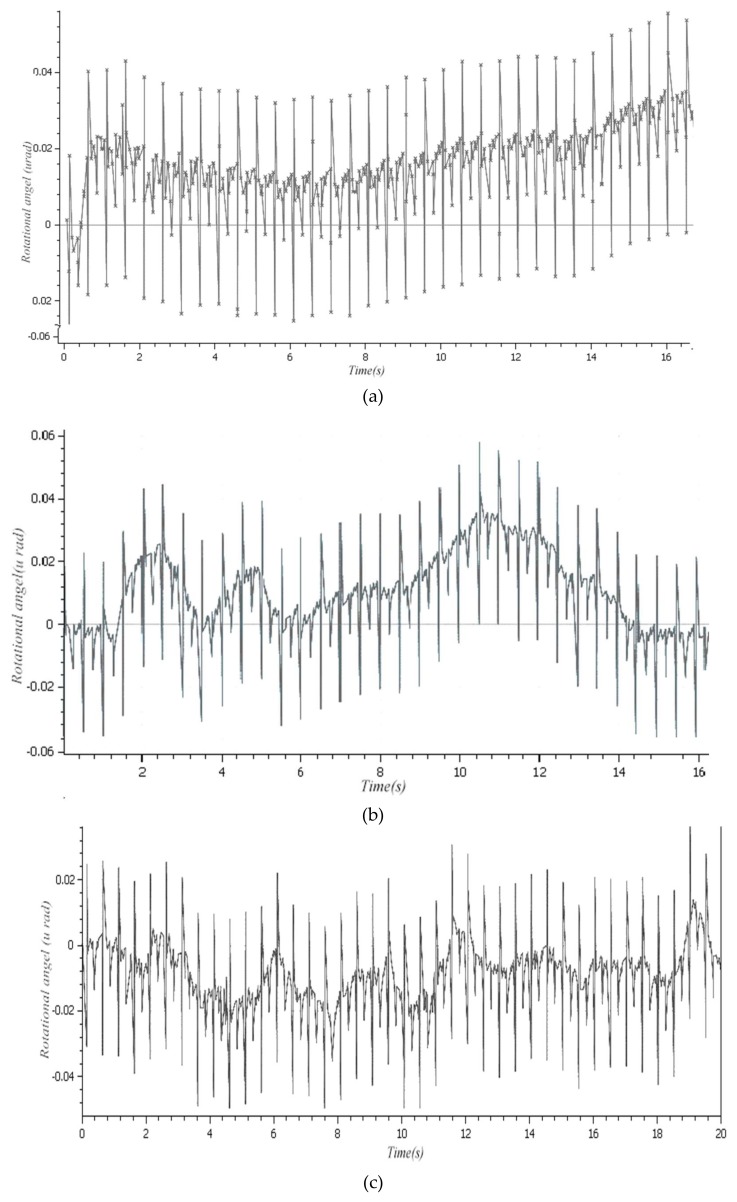
Displacement of three rotational directions along x−y−z axes; (**a**) Around x-axis, (**b**) Around y-axis, (**c**) Around z-axis.

A double frequency laser interferometer was used to obtain the precise position of three rotational directions x−y−z, the displacement step was 33.5 nm, and the initial voltage of the PZT actuators was 20 V. The measurement results are shown in Figure 11a–c, respectively.

The comparison of the kinematic results between the simulation and experiment is given in Table 4.

It can be seen that the experimental results are not compatible with the theoretical simulation results due to the three actuators mounted during the 3D printing process, consistency of surface shape, and so on. Therefore, in the processing of the spatially compliant mechanism, it is necessary to take into account the shape error of its topological configuration.

### 5.5. Modal Analysis of the First Three Orders

**Figure 12:**
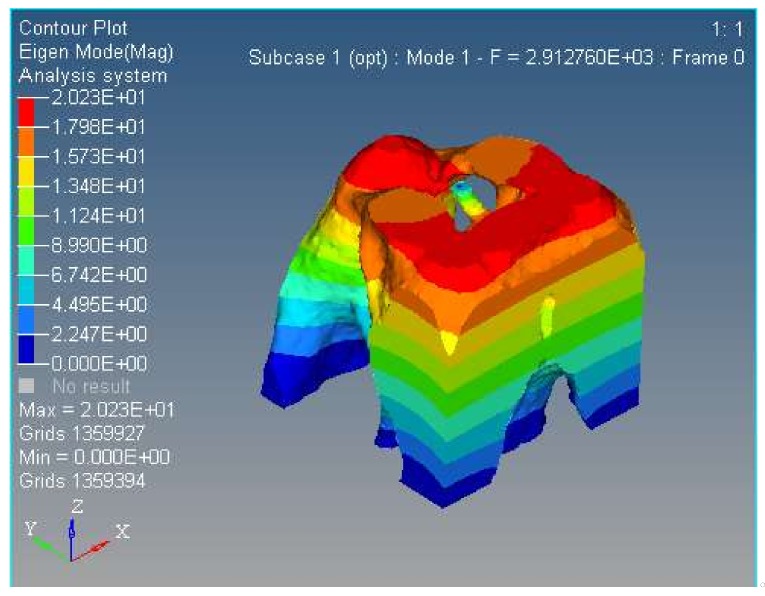
First order modal of the proposed spatially compliant mechanism with three-rotational-DOFs.

**Figure 13:**
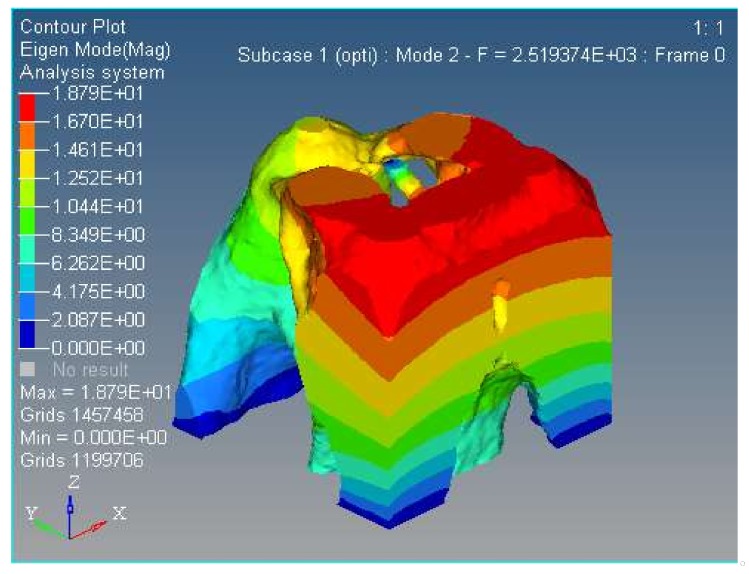
Second order modal of the proposed spatially compliant mechanism with three rotational DOFs.

Modal analysis is inherent to the vibration characteristics of mechanical structures, and it is important in the micro/nano manufacturing field. The Optistruct software was used to analyze the first and second modal of the spatially compliant mechanism proposed in this paper, and modal analysis is proposed for the same input load conditions and boundary constraint conditions. The first and second order modal analyses are shown in Figure 12 and Figure 13, respectively.

The results show that the natural frequency is higher than the same compliant mechanism with the hinge replacement method proposed in the literature [17].

## 6. Conclusions

In this paper, a topology optimization approach was proposed for the design of three rotational degree-of-freedom (DOF) spatially compliant mechanisms. In this method, the isomorphic Jacobian matrix of the 3-UPC type parallel prototype manipulator was calculated, and then the orthogonal triangular decomposition and differential kinematic method were applied to uncouple the Jacobian matrix in order to construct a constraint field of topology optimization. Firstly, in terms of the 3-UPC type parallel prototype manipulator, the Jacobian matrix was derived to map the inputs and outputs to be used during the topology optimization process. Secondly, orthogonal triangular decomposition with the differential kinematic method was used to reconstruct the uncoupled mapping matrix for the topology optimization process. Finally, the SIMP method was applied to construct the topological model in combination with the isomorphic mapping matrix. A typical three rotational DOF spatially compliant mechanism was used as a numerical example to demonstrate the effectiveness of the proposed method.

(1) In order to build accurate modeling after the structural topology optimization, the SIMP topology optimization method combined with the isomorphic mapping matrix of 3-UPC type parallel prototype manipulator was used for the structural synthesis for the spatially compliant mechanism with three rotational DOFs.
(2) The optimized three-DOF spatially compliant mechanism with the isomorphic mapping matrix was imported to Hyperworks for finite element static analysis. The maximum and minimum rotational angles of 0.47 × 10^−4^ rad and −0.21 × 10^−4^ rad in *x* direction, 0.51 × 10^−4^ rad and −0.46 × 10^−4^ rad in *y* direction, and 0.12 × 10^−4^ rad and −0.48 × 10^−5^ rad in *z* direction were obtained by simulation, while the corresponding experimental rotational angles of the maximum and minimum were 0.36 × 10^−4^ rad and −0.18 × 10^−4^ rad in *x* direction, 0.39 × 10^−4^ rad and −0.12 × 10^−4^ rad in *y* direction, and 0.18 × 10^−4^ rad and −0.32 × 10^−4^ rad in *z* direction, respectively.
(3) The comparative simulation studies, the stress distribution and the first/second order modal of the proposed spatially compliant mechanism, show that the proposed method can ensure the integral isomorphism characteristics between the proposed spatially compliant mechanism and the conventional parallel prototype manipulator with uniform stiffness and vibration suppression.
(4) The compliant mechanism obtained through the topology optimization had a very irregular shape, which is related to the configuration of the topological optimization conditions. By using a topological optimization method combining a Jacobian matrix with the SIMP method of isomorphism mapping, regardless of the initial optimization conditions, the resulting topologies all had the same differential motion characteristics as the parallel prototype mechanism, i.e., isomorphism. The piezoelectric actuator is in an enclosed space and its spatial configuration is determined based on the parallel prototype mechanism actuator configuration. The approach adopted consisted of interrupting the printing process during 3D printing and resuming it once the piezoelectric actuator is implanted. Therefore, there is no better way to implement the driver than the 3D printing technology.

## Figures and Tables

**Table 1:** Structural parameters of the 3-UPC type parallel prototype manipulator.

Structural Parameters	Values	Output	Values
Length of moving platform	b=100 mm	Actuator 1	$\Delta l_{1}=0.03\sin\left(3t\right)$
Length of fixed platform	a=150 mm	Actuator 2	$\Delta l_{2}=0.05\sin\left(3t\right)$
Height	h=200 mm	Actuator 3	$\Delta l_{3}=0.025\sin\left(3t\right)+3$

**Table 2:** Stress distribution before/after topology optimization.

	Max	Min
Before	8.769 × 10^−2^ MPa	2.152 × 10^−6^ MPa
After	8.752 × 10^−2^ MPa	2.983 × 10^−13^ Mpa

**Table 3:** The parameters of the piezoelectric (PZT) actuator.

Terms	Values
Model of PZT	PSt150 VS10
Nominal stroke (μm)	9 ± 10%
Stiffness (N/μm)	50 ± 20%
Nominal pull/thrust (N)	550/100
Electrostatic capacity (μF)	0.35 ± 20%
Resonant frequency (kHz)	40
Length (mm)	19 ± 0.3
Driving voltage (V)	0–120

**Table 4:** Comparison of the kinematic results between the simulation and experiment.

Direction	Simulation (maximum)	Experiment (maximum)	Simulation (minimum)	Experiment (minimum)
Rotation around x-axis	0.47 × 10^−4^ rad	0.36 × 10^−4^ rad	−0.21 × 10^−4^ rad	−0.18 × 10^−4^ rad
Rotation around y-axis	0.51 × 10^−4^ rad	0.39 × 10^−4^ rad	−0.46 × 10^−4^ rad	−0.12 × 10^−4^ rad
Rotation around z-axis	0.12 × 10^−^^4^ rad	0.18 × 10^−^^4^ rad	−0.48 × 10^−5^ rad	−0.32 × 10^−^^4^ rad
